# Assessment of Tobacco-Related Approach and Attentional Biases in Smokers, Cravers, Ex-Smokers, and Non-Smokers

**DOI:** 10.3389/fpsyg.2016.00172

**Published:** 2016-02-26

**Authors:** Marcella L. Woud, Joyce Maas, Reinout W. Wiers, Eni S. Becker, Mike Rinck

**Affiliations:** ^1^Department of Psychology, Mental Health Research and Treatment Center, Ruhr-Universität BochumBochum, Germany; ^2^Behavioural Science Institute, Radboud University NijmegenNijmegen, Netherlands; ^3^Addiction Development and Psychopathology Lab, University of AmsterdamAmsterdam, Netherlands

**Keywords:** tobacco dependence, approach-avoidance, attention, AAT, SRC, STIAT, Stroop

## Abstract

According to theories of addictive behaviors, approach and attentional biases toward smoking-related cues play a crucial role in tobacco dependence. Several studies have investigated these biases by using various paradigms in different sample types. However, this heterogeneity makes it difficult to compare and evaluate the results. The present study aimed to address this problem, via (i) a structural comparison of different measures of approach-avoidance and a measure of smoking-related attentional biases, and (ii) using within one study different representative samples in the context of tobacco dependence. Three measures of approach-avoidance were employed: an Approach Avoidance Task (AAT), a Stimulus Response Compatibility Task (SRC), and a Single Target Implicit Association Test (ST-IAT). To assess attentional biases, a modified Stroop task including smoking-related words was administered. The study included four groups: *n* = 58 smokers, *n* = 57 non-smokers, *n* = 52 cravers, and *n* = 54 ex-smokers. We expected to find strong tobacco-related approach biases and attentional biases in smokers and cravers. However, the general pattern of results did not confirm these expectations. Approach responses assessed during the AAT and SRC did not differ between groups. Moreover, the Stroop did not show the expected interference effect. For the ST-IAT, cravers had stronger approach associations toward smoking-related cues, whereas non-smokers showed stronger avoidance associations. However, no such differences in approach-avoidance associations were found in smokers and ex-smokers. To conclude, these data do not provide evidence for a strong role of implicit approach and attentional biases toward smoking-related cues in tobacco dependency.

## Introduction

I need more cigarettes

Give me more cigarettes, I need

Gotta get more cigarettes

I want more cigarettes

Cigarettes

Cigarettes

(More Cigarettes–Replacements–2012)

The inability to control drug use is a hallmark symptom of a drug addiction (Diagnostic Statistical Manual of Mental Disorders; DSM-5; 2013). Smoking, for example, represents such an addictive behavior, and it is considered one of the most difficult addictions to break. According to the World Health Organization (WHO) report in 2008, tobacco smoking causes 5.4 million deaths per year, and it remains the leading preventable cause of death worldwide (World Health Organization, 2008). Furthermore, research also showed that smoking increases the risk of engaging in other addictive behaviors (Merrill et al., 1999; Creemers et al., 2009). Hence, it is not surprising that there is a growing interest to elucidate the motivational and reward mechanisms underlying this destructive behavior.

According to dual process models of addiction (e.g., Deutsch and Strack, 2006; Wiers et al., 2007; Gladwin et al., 2011; for critical discussion see Gladwin and Figner, 2014), addictive behaviors can be understood best as the output of two distinct types of processes. On the one hand, reflective processes with limited (cognitive) capacity involve processes that are slower, more deliberate and explicit. On the other hand, impulsive processes do not require limited (cognitive) capacity and involve processes that are fast and automatic. It has been suggested that the latter processes are particularly involved in emotional and motivational aspects of behavior. Such dual process models of addiction posit that addictive behaviors are the result of an imbalance between these two processes, i.e., there is no cooperative interplay: There are easily activated, drug-oriented impulsive processes, in combination with relatively slow reflective processes that are not strong enough to control or regulate the impulsive process. Furthermore, and in line with the incentive-sensitization-theory of Robinson and Berridge (1993, 2003, 2008), dual process models of addiction hypothesize that the impulsive processes become sensitized with repeated drug use. Drug-related cues acquire incentive salience, which results in an activation of the mesolimbic dopamine system and an increase in dopamine levels. As a consequence of this neurological chain, the brain “interprets” drug-related cues as rewarding cues, and therefore prepares the corresponding motivational state, i.e., an approach action tendency, aimed at consuming the drug of interest. From an information processing perspective, this explains behavioral phenomena such as attentional and approach biases for drug-related cues: Due to the incentive salience of these cues, they automatically capture an individual's attention and activate approach-related behaviors.

Over the last decades, there has been a surge of interest in tobacco-related information processing biases (for an overview and meta-analysis, see e.g., Waters and Sayette, 2006; Field and Cox, 2008; Rooke et al., 2008). Such investigations are important from a theoretical but also from a clinical perspective: On the one hand, such studies can test specific hypotheses derived from models of addiction, and on the other hand, these studies can advance our understanding of factors related to the high number of relapse in tobacco dependence. For example, Waters et al. (2003) found that smokers who showed a greater attentional bias for smoking-related words were more likely to lapse in the short-term.

Before summarizing studies investigating tobacco-related approach biases, an important distinction has to be made. This distinction concerns the operationalization of approach biases, namely whether they are operationalized as symbolic or actual motor responses. Regarding the assessment of symbolic motor responses, the Stimulus-Response Compatibility (SRC, Mogg et al., 2003) task has been used to assess symbolic tobacco-related approach biases. During the SRC, participants are instructed to move a manikin figure toward (approach) or away (avoidance) from, for example, smoking-related or neutral pictures. The time needed to initiate the manikin's approach and avoidance movements serves as the dependent variable. Studies employing the SRC showed that smokers are faster to approach than to avoid smoking-related cues (e.g., Mogg et al., 2003, 2005; Bradley et al., 2004, 2008; Thewissen et al., 2007). Regarding the assessment of actual motor responses, the Approach-Avoidance Task (AAT; Rinck and Becker, 2007) is a suitable paradigm. Indeed, it also has been used to assess tobacco-related approach biases. During the AAT, participants are instructed to pull (approach) and to push (avoidance) a joystick in response to, for example, smoking-related or neutral pictures, that appear on the computer screen. Here, the time needed to execute the push and pull movements serve as the dependent variable. Most AATs apply an indirect task version. That is, the instructions do not ask participants to respond to the pictures' content. Instead, participants are required to respond to an unrelated feature such as the pictures' orientation or format. The advantage of such an indirect task version is that participants respond to a stimulus feature that is independent of the stimulus dimension that the task aims to assess, which disguises the research question and makes the use of response strategies less likely (Rinck and Becker, 2007). In the context of tobacco dependence, the AAT is a rather novel paradigm, and to the best of our knowledge, only three studies have employed the AAT so far (but for more AAT studies in the context of alcohol dependency, see e.g., Palfai and Ostafin, 2003; Wiers et al., 2010, 2011; Eberl et al., 2013; Kersbergen et al., 2015). The study by Wiers C. E. et al. (2013), examined tobacco-related approach biases in heavy smokers, non-smokers, and ex-smokers. Results showed that heavy smokers were faster to approach smoking-related pictures compared to non-smokers and ex-smokers. Moreover, this approach bias was correlated with levels of craving. The study by Machulska et al. (2015) compared smokers to non-smokers, and found that smokers, unlike non-smokers, exhibited an approach bias toward smoking-related pictures compared to food-related control pictures (see also Larsen et al., 2014). Finally, according to results of Watson et al. (2013), tobacco-related approach biases can also be conditional. They tested a group of deprived cigarette smokers and found that the bias assessed at baseline was associated with participants' level of craving. After the baseline assessment, half of the participants were allowed to smoke a cigarette. These participants reported a reduction in craving but an increase in approach bias.

Beyond the studies examining actual and symbolic tobacco-related approach biases, there are also studies targeting tobacco-related approach associations. Word categorization tasks such as the Implicit Association Test (IAT; Greenwald et al., 1998) have been employed in this type of research. During the IAT, participants simultaneously categorize target stimuli (e.g., smoking-related vs. control stimuli) and attribute words (e.g., approach- or avoidance-related words) as fast as possible into the appropriate superordinate category. The difference in reaction times between the possible combinations (e.g., smoking-related stimuli and approach attributes share the same response key, and neutral stimuli and avoidance-related words share the same response key) is assumed to reflect whether smoking is associated more strongly with either attribute category, with relatively fast responses reflecting relatively strong associations. The study by De Houwer et al. (2006) examined such associations and found that smokers indeed had stronger approach- than avoidance-related tobacco associations, respectively. However, most of the IAT studies compared general positive vs. negative smoking-related associations, and here the evidence is less clear (e.g., Swanson et al., 2001; Sherman et al., 2003; Huijding et al., 2005).

Regarding tobacco-related attentional biases, several studies found that smokers are slower to respond to smoking-related pictures (visual probe task) and words (Stroop task), compared to neutral pictures or words (visual probe task: e.g., Mogg et al., 2003, 2005; Bradley et al., 2008; Stroop task: e.g., Munafò et al., 2003, 2005; Larsen et al., 2014; and for a meta-analysis, see Cox et al., 2006). There is also evidence which further specifies these findings. Results of Mogg and Bradley (2002) showed a positive correlation between smoking-related attentional biases and daily cigarette consumption. Moreover, Wertz and Sayette (2001) found a greater Stroop interference in participants who were told that they were allowed to smoke during the study, compared to those who were told they were not allowed to smoke. Finally, smoking-related attentional biases seem to be related to levels of self-reported craving (Zack et al., 2001; Mogg and Bradley, 2002), and increase after participants have been deprived of smoking (Cox et al., 2006). For example, using a visual probe task, Field et al. (2004) found that deprived smokers maintained their gaze toward smoking-related cues compared to neutral cues.

In summary, there is evidence showing that tobacco dependency is characterized by smoking-related approach and attentional biases. Despite the importance of these findings, however, there are two significant limitations: First, within previous studies, only a limited number of groups have been compared. Second, previous studies employed only a limited number of tasks. This heterogeneity makes it difficult to compare and evaluate these studies, particularly in relation to the underlying theory. The present study aimed to address this problem via (i) a structural comparison of different measures of approach-avoidance and the most commonly used paradigm to assess attentional biases (i.e., the Stroop), and (ii) using within one study different representative samples in the context of tobacco dependence. To assess smoking-related approach avoidance biases, three different measures were used: the Approach Avoidance Task (AAT), the Stimulus Response Compatibility Task (SRC), and a Single Target Implicit Association Test (STIAT; Wigboldus et al., 2004). Given the fact that smoking does not have an inherently meaningful contrast category (such as, for example, alcohol vs. soft drinks), we chose to use a STIAT instead of an IAT. The AAT and SRC used pictorial stimuli (smoking-related and matched control pictures), the STIAT used word stimuli (targets: smoking-related words, attributes: approach avoidance words). A modified Stroop including smoking-related words was administered to assess tobacco-related attentional biases. Finally, we also assessed explicit attitudes toward smoking and levels of craving over the course of the study. The study included four groups: smokers, cravers, ex-smokers, and non-smokers. Following the predictions of theories of addictive behaviors and the existing empirical evidence in this context, our main hypothesis was to find strong tobacco-related approach and attentional biases in smokers and cravers, compared to ex-smokers and non-smokers. Moreover, we expected that tobacco-related approach and attentional biases would be correlated positively across smokers, cravers, and ex-smokers.

## Methods

### Participants

A total of 232 students from Radboud University (NL) were tested (*M*_age_: 22.36, *SD* = 3.2, 158 females). Within this group, there were *n* = 59 smokers, *n* = 59 non-smokers, *n* = 56 cravers, and *n* = 58 ex-smokers. The selection criteria were as follows: Smokers were included if they were smoking at least six cigarettes a day for at least 2 months. The same criteria applied for cravers. In order to avoid craving, smokers were instructed to smoke a cigarette prior to the study. However, cravers were instructed to not smoke for 6 h prior to the study. The group of non-smokers included individuals who had never smoked a cigarette or a joint. Ex-smokers were included if they had stopped smoking at least 6 months earlier and had smoked a minimum of six cigarettes a day while actively smoking. Prior to the analyses, 11 participants were excluded: Two non-smokers (one because of technical problems during testing and another who was actually smoking once in a while), four cravers (one did not smoke six or more cigarettes a day, one was tested too early and thus did not crave for 6 h, and two did not comply with the rule to not smoke for 6 h prior to the study), four ex-smokers and one smoker (technical problems during testing), leaving a total sample of *N* = 221 (*n* = 58 smokers, *n* = 57 non-smokers, *n* = 52 cravers, and *n* = 54 ex-smokers)^1^.

### Materials

#### Self-report measures

##### Fagerström test for nicotine dependence (FTND)

The FTND (Heatherton et al., 1991) is a self-report measure assessing the degree of nicotine dependence. It contains six items, e.g., “How many cigarettes per day do you smoke?” “How soon after waking do you smoke your first cigarette?” The higher the FTND sum score, the higher participants' level of dependence.

##### Explicit attitudes toward smoking

To assess explicit attitudes toward smoking, participants were asked to evaluate eight adjective pairs (e.g., smoking is “good–bad,” “sociable–unsociable,” “sexy–unsexy”) on a 7-point scale (see Huijding et al., 2005; Huijding and de Jong, 2006).

##### Pictorial stimuli

The pictorial stimuli included 20 smoking-related pictures and 20 matched control pictures (for examples, see Supplementary Material). These 40 pictures were divided across two sets, i.e., set A and B, each containing 10 smoking-related pictures and the corresponding 10 matched control pictures.

##### Approach avoidance task (AAT)

During the AAT (Rinck and Becker, 2007), participants responded to pictures presented on the computer screen by approaching and avoiding them using a joystick. The joystick was positioned in front of the computer screen, tightly fastened to the table. The instructions said that all pictures were tilted either slightly to the left or right, and that the tilt determined whether the pictures had to be pulled (approach movement) or pushed (avoidance movement; for a similar procedure, see Cousijn et al., 2011). Within each of the four participants groups, half of the participants pulled left-tilted and pushed right-tilted pictures, whereas the other half pushed left-tilted and pulled right-tilted pictures. Participants initiated each trial by pressing a button of the joystick with their index finger while holding the joystick in the central position. When the picture appeared, participants had to decide quickly whether the picture was tilted to the left or to the right, and had to respond according to their instructions. During pushing, the pictures became smaller, whereas they became larger during pulling. This zoom supported the approach-avoidance effect visually. Moreover, participants were instructed to “pull the joystick toward themselves,” and to “push it away from them.” Via these instructions, the movements' reference point was the participant's body. This disambiguated the movements, and labeled them as clear and unambiguous approach or avoidance movements. After pushing or pulling the joystick all the way into the right direction, participants had to bring it back to the central position and start the next trial. Pictures disappeared only when the joystick was pulled or pushed in the correct direction and when the joystick was pulled or pushed by an angle of 30 degrees.

The AAT started with a practice block during which two practice pictures were pushed and pulled 10 times each. After that, 160 assessment trials followed, including 10 smoking-related pictures and 10 matched control pictures. The assessment was divided into two blocks of 80 trials each. Within each block, the smoking-related pictures and matched control pictures were pushed and pulled four times each [i.e., (4 × 10)+(4 × 10) = 80 × 2 = 160 trials in total].

##### Stimulus response compatibility task (SRC)

In each trial of the SRC task (Mogg et al., 2003), a picture appeared in the center of the screen. In addition, a manikin figure was displayed either below or above the picture. Participants were instructed to move the manikin figure either toward or away from the picture by making use of the keys “2” (manikin moved downwards) and “8” (manikin moved upwards) on the numeric part of the keyboard. There were two blocks with two different stimulus-response assignments: One block required participants to move the manikin toward smoking-related pictures (approach movement) and to move the manikin away from control pictures (avoidance movement), whereas the other block required participants to move the manikin away from smoking-related pictures and toward control pictures. For the sake of brevity, the following terms will be used to describe these two different stimulus-response assignments: compatible block: manikin approaches smoking-related pictures and avoids control pictures; incompatible block: manikin avoids smoking-related pictures and approaches control pictures. The latency between picture onset and the participant's response served as the dependent variable. All participants completed both blocks. However, the order of blocks was counterbalanced: Within each of the four participants groups, half of the participants started with the compatible block and then completed the incompatible block, whereas the other half started with the incompatible block and then completed the compatible block. Within each block, the manikin appeared below the picture in 50% of the trials, and above the picture in the other 50%. When the manikin appeared below the picture, 50% of the trials required a down response, whereas the other 50% required an up response, and the same was true when the manikin appeared above the picture. The manikin position and picture type varied randomly over trials.

The SRC started with a practice block during which the manikin approached one picture four times and also avoided one picture four times. After that, 160 assessment trials followed, including 10 smoking-related pictures and 10 matched control pictures. The assessment was divided in two blocks of 80 trials each. Within each block, the smoking-related pictures and matched control pictures were approached and avoided four times each [i.e., (4 × 10)+(4 × 10) = 80 × 2 = 160 trials in total].

##### Single target implicit association test (STIAT)

The STIAT (Wigboldus et al., 2004) consisted of a complete sequence of five blocks: (a) attribute discrimination, (b) practice combined block, (c) first combined block, (d) practice reversed combined block, and (e) reversed combined block. Each block started with instructions describing the discrimination category and the assignment of the response keys (left vs. right). The procedure started with (a) the attribute discrimination block, in which participants had to sort words that belonged to two categories, namely approach or avoidance. Participants were asked to press one key in response to approach-related words, and the other key in response to avoidance-related words (i.e., either key “A” on the very left part of the keyboard or key “6” on the numeric part of the keyboard). The stimuli in this block consisted of six approach-related attribute words and six avoidance-related attribute words. Words were presented one after another in a fixed random order. In the second block, six smoking-related target words were also presented. Participants therefore practiced the combined block (b). There were two different response assignments: one assignment required participants to categorize smoking-related target words with the same key as approach-related attribute words. The other assignment required participants to categorize smoking-related target words with the same key as avoidance-related attribute words. For the sake of brevity, the following terms will be used to describe these two response assignments: compatible block: smoking-related targets and approach-related attributes shared the same response key; incompatible block: smoking-related targets and avoidance-related attributes shared the same response key. The combined practice block included 24 trials: The six target words were presented once, the six attribute words that required the same response were also shown once, and the six words that required the opposite response key were presented 12 times. Because targets were assigned to only one key during combined blocks, there are fewer responses on the opposite key. Hence, to balance this mismatch of responses by the left and right key, attributes assigned to the opposite side of the targets were presented twice as often, resulting in an equal number of left and right key responses in each of the combined blocks. The key-assignment was counterbalanced within each of the four participant groups: Half of the participants were told to press the left key (“A” key) in response to all targets, and the other half were told to press the right key (“6” key) in response to all targets. Moreover, we controlled for the sequence of the combined blocks: Within each of the four participants groups, half of the participants started with the compatible block and then completed the incompatible block, whereas the other half started with the incompatible block and then completed the compatible block.

After the practice trials, the actual combined block followed (c). This block included 72 trials: The six target words were presented three times (18 trials), the six attribute words which required the same response were also shown three times (18 trials), and the six words which required the opposite response were presented six times each (36 trials). Next, participants practiced the reversal of the response assignment for target words (d). That is, participants who had pressed the approach key in response to smoking-related targets now had to respond with the avoidance key, the other half of the participants vice versa. This combined reversed practice block also consisted of 24 trials: The six target words were presented once, the six attribute words that required the same response were also shown once, and the six words that required the opposite response were presented 12 times each. Finally, the actual reversed combined block followed (e). This block included 72 trials again: The six target words were presented three times, the six attribute words which required the same response were also shown three times, and the six words which required the opposite response were presented six times. During each trial, reminder labels (appropriate category names positioned in the top left and top right corner of the screen) remained visible. Within each block, stimuli appeared in the same fixed random order for each participant. After incorrect responses, a red “X” appeared in the center of the screen. Given the high numbers of German students at Radboud University, we had two STIATs; a Dutch and a German version.

##### Emotional stroop

During this task, participants categorized word stimuli according to their print color. The stimuli were presented on cards. There were five print colors: white, blue, red, green, and yellow. There were three types of cards. All participants started with the practice card. Here, meaningless colored strings of “XXX” were presented. After that, the smoke card or the neutral card was presented (randomized). On the smoke card, eight smoking-related words were shown (e.g., cigarette, smoke, cigar). These words differed from the smoking-related words used during the STIAT. On the neutral card, eight household-related words were shown (e.g., towel, broom, spoon). The order of the smoke and the neutral card was random. All cards contained 40 stimuli each, i.e., eight stimuli distributed across five columns. Each card appeared on the screen after a mouse click initiated by the experimenter. As soon as the participant had named the last word's print color, the experimenter clicked again and the card disappeared. Reaction times were saved on the computer, for each card separately, and these reaction times were used in the analyses. Participants' errors were recorded by the experimenter, who was blind to the type of card that was presented. Given the high numbers of German students at Radboud University, we used a German and a Dutch Stroop version.

##### Procedure

Participants were tested individually in separate testing cubicles. After having signed informed consent, participants' level of carbon monoxide (CO) was assessed by means of the piCO+ smokelyzer (Bedfont Scientific, Kent, England). For smokers, CO levels were assessed 10 min after they had smoked their cigarette. Next, smokers, cravers and ex-smokers answered a question about their level of craving (“How strong is your urge to smoke a cigarette right now?”) using a scale from 0 (= no urge) to 100 (= strong urge). Moreover, smokers and cravers had to indicate how many cigarettes they would smoke on a normal day. Ex-smokers were asked to indicate this for the time they were still smoking. Then, the four computer tasks followed. There were two orders and this was counterbalanced: Within each of the four participants groups, half of the participants received order one (STIAT, SRC, Stroop, AAT), the other half order two (AAT, Stroop, SRC, STIAT). The tasks' order was linked to the picture set (A or B, example: if a participant started with the AAT, picture set A was used for the AAT, and picture set B was used for the SRC, and vice versa if a participant started with the SRC). That is, for task order one, the AAT always included picture set A and the SRC included picture set B. For task order two, the AAT always included picture set B and the SRC included picture set A. After the computer tasks, smokers, cravers and ex-smokers completed a second craving question and the FTND. Ex-smokers received an adapted version of the FTND that was related to their past smoking behavior. The smoking attitude rating was then completed by all participants. Finally, cravers were asked to smoke a cigarette and after 10 min, their CO levels were assessed a second time. This second assessment served as an extra check for the cravers' temporal abstinence, i.e., we expected their CO value to be higher than their CO value assessed before the start of the study. The present study had the necessary ethical approvals via the Behavioural Science Institute.

## Results

### Participant characteristics

Table 1 gives an overview of the samples' characteristics and the means and standard deviations of the following measures: average of daily smoked cigarettes, levels of carbon monoxide (CO) pre study, craving pre and post-study, and scores on the Fagerström Test for Nicotine Dependence (FTND). A chi-square test revealed that the four groups did not differ concerning gender, $\chi_{\left(3\right)}^{2}=4.84$, *p* = 0.18. Univariate ANOVAs were conducted to examine the following baseline measures (please note that not all groups were involved in all comparisons): Age, *F*_(3, 217)_ = 7.81, *p* < 0.001, eta^2^ = 0.1; Average of daily smoked cigarettes, *F*_(2, 161)_ = 0.51, *p* = 0.6; CO levels pre study, *F*_(3, 202)_ = 94.91, *p* < 0.001, eta^2^ = 0.59; Craving pre study, *F*_(2, 160)_ = 91.2, *p* < 0.001, eta^2^ = 0.53; FTND scores, *F*_(2, 161)_ = 7.84, *p* < 0.01, eta^2^ = 0.09.

**Table 1:** **Descriptives of the four groups**.

	**Gender: f/m**	**Age**	**Average daily smoking**	**CO levels pre**	**CO levels post**	**Craving pre study**	**Craving post-study**	**FTND**
**Group**		**M (SD, n)**	**M (SD, n)**	**M (SD, n)**	**M (SD, n)**	**M (SD, n**	**M (SD, n**	**M (SD, n)**
Smokers	39/19	22.28 (2.55, 58)	12.59 (4.83, 58)	17.02 (8.65, 58)		20.37 (23.97, 57)	31.75 (25.5, 57)	5.26 (1.63, 58)
Cravers	31/21	22.88 (3.72 52)	12.94 (6.2, 52)	7.02 (5.3, 52)	11.16 (6.01, 49)	58.98 (20.83, 52)	65.63 (22.28, 52)	5.38 (1.33, 52)
Ex-smokers	37/17	23.57 (3.59, 54)	13.70 (6.69, 54)	2.41 (1.12, 39)		10.1 (11.09, 52)	13.08 (17.41, 52)	4.43 (1.06, 54)
Non-smokers	45/12	20.88 (2.23, 57)		1.77 (0.82, 57)				

CO levels pre: Levels of carbon monoxide (CO) assessed before the study (please note Footnote 1); CO levels post: Levels of carbon monoxide (CO) assessed again in cravers after the study; Average daily smoking: Average of daily smoked cigarettes for smokers, cravers and ex-smokers (retrospective); Craving pre study: Levels of cigarette craving in smokers, cravers and ex-smokers before the study; Craving post-study: Levels of cigarette craving in smokers, cravers and ex-smokers after the study; FTND: Mean sum score of the Fargerström Test for Nicotine Dependence (FTND) in smokers, cravers, and ex-smokers (retrospective).

These outcomes were treated as follows: For age, we repeated the main analyses (i.e., for the AAT, SRC, STIAT, and Stroop) including age as a covariate. This did not change the results, and thus for clarity and given the lack of specific hypotheses regarding age, we report unadjusted analyses without this covariate, and did not analyse this baseline imbalance further. Hence, for the sake of brevity, we report all analyses without this factor. Moreover, we did not further analyze FTND scores, given the fact that the ex-smokers' score is a retrospectively assessed score and thus not an optimal measure. However, we did further examine the findings concerning the CO levels and craving scores pre study. Regarding the pre study CO levels, Bonferroni *post-hoc* tests including all four groups (i.e., smokers, non-smokers, cravers, ex-smokers) revealed that all group comparisons were significant (*p's* < 0.002), except for the non-smokers vs. ex-smokers comparison (*p* = 1). Regarding the craving scores pre study, Bonferroni *post-hoc* tests including smokers, cravers, and ex-smokers revealed significant differences for all three comparisons, *p's* < 0.03.

### Craving over the course of the study

We also assessed participants' level of craving over the course of the study. A repeated-measures ANOVA including the between-subjects factor Group (smokers, cravers, ex-smokers) and the within-subjects factor Time (craving pre, craving post) revealed a significant main effect of Time, *F*_(1, 158)_ = 23.88, *p* < 0.001, eta^2^ = 0.13, and Group, *F*_(2, 158)_ = 99.91, *p* < 0.001, eta^2^ = 0.56. Moreover, there was a marginally significant Time x Group interaction; *F*_(2, 158)_ = 2.79, *p* = 0.065, eta^2^ = 0.03. This interaction was further examined by three paired-samples *t*-tests, i.e., one for each group comparing craving scores pre vs. post: smokers: *t*_(56)_ = 3.86, *p* < 0.001; cravers: *t*_(51)_ = 2.76, *p* < 0.01; ex-smokers: *t*_(51)_ = 1.92, *p* = 0.06. Following this, smokers' and cravers' level of craving significantly increased over the course of the study. In the group of ex-smokers, this increase was marginally significant, although it would no longer be after application of a Bonferroni correction (for means and standard deviations, see Table 1).

### Analyses approach-avoidance biases

For the analyses of the AAT, SRC, and STIAT, the effects of potential outliers were corrected by computing the median reaction time (RT) of each participant. Thus, the means reported below are means of medians.

### Approach avoidance task (AAT)

The analysis included only trials during which a participant pushed or pulled the joystick all the way into the right direction within one movement. As a first step, we examined the groups' error trials by means of a univariate ANOVA. Results showed that the groups did not differ here: *F*_(3, 217)_ = 1.64, *p* = 0.18 (smokers: *M* = 0.05, *SD* = 0.04; non-smokers: *M* = 0.05, *SD* = 0.05; cravers: *M* = 0.07, *SD* = 0.09; ex-smokers: *M* = 0.05, *SD* = 0.06).

Next, a difference score per participant was calculated. As a first step, RTs of pull movements were subtracted from RTs of push movements, for both picture types (i.e., smoking and control). As such, a positive difference score reflects an approach bias. After that, we subtracted the control pictures' difference score from that of smoking-related pictures. Here, a positive difference score indicates a stronger approach bias toward smoking-related pictures. Finally, participants with an error percentage greater than 20% were excluded from the analysis (non-smokers *n* = 2, cravers *n* = 3, ex-smokers *n* = 2).

To analyze the AAT data, a univariate ANOVA was conducted with Group (smokers, non-smokers, cravers, ex-smokers) and Order Tasks (one, two) as between-subjects factor, and the overall difference score as dependent variable. Of most interest was the main effect of Group. However, this effect did not reach significance, *F*_(3, 206)_ = 0.9, *p* = 0.44. As such, the groups did not differ in their approach-avoidance responses toward smoking-related and control pictures (for an overview of means, standard deviations and *n*'s per group, see Table 2)^2^.

**Table 2:** **Means and standard deviations for all four computer tasks per group**.

**Task**	**Group**	**Score 1 *M* (*SD*)**	**Score 2 *M* (*SD*)**	**Overall difference score *M* (*SD*)**	***n***
AAT	Smokers	−17 (78)	−14 (65)	−3 (76)	58
	Cravers	−0.7 (54)	−19 (48)	19 (61)	49
	Ex-smokers	−13 (69)	−17 (60)	4 (69)	52
	Non-smokers	−14 (76)	−18 (60)	4 (83)	55
SRC	Smokers	760 (146)	843 (169)	83 (114)	56
	Cravers	796 (119)	901 (221)	106 (147)	52
	Ex-smokers	838 (179)	887 (222)	49 (143)	53
	Non-smokers	770 (121)	823 (156)	52 (104)	57
STIAT	Smokers	588 (89)	594 (74)	6 (92)	57
	Cravers	594 (87)	641 (100)	47 (86)	52
	Ex-smokers	614 (85)	623 (79)	9 (103)	53
	Non-smokers	594 (78)	577 (64)	−17 (60)	56
Stroop	Smokers	27898 (4829)	26411 (4434)	1487 (2634)	57
	Cravers	31644 (7757)	29116 (4398)	2528 (6965)	46
	Ex-smokers	28224 (4858)	26437 (4853)	1787 (2634)	50
	Non-smokers	31601 (12206)	29537 (9761)	2064 (15419)	55

AAT, Approach Avoidance Task; Score 1, RTs push movements—RTs pull movements smoke pictures; Score 2, RTs push movements—RTs pull movements control pictures; Overall difference score, Score 1—Score 2, i.e., a positive difference score indicates a stronger approach bias toward smoking-related pictures. SRC: Score 1, Compatible block (manikin approaches smoking-related pictures and avoid control pictures); Score 2, Incompatible block (manikin avoids smoking-related pictures and approaches control pictures); Overall difference score, Incompatible block—compatible block, i.e., a positive difference score indicates faster approach of smoking-related pictures. STIAT: Score 1, Compatible block (smoking-related targets and approach-related attributes shared the same response key); Score 2, Incompatible block (smoking-related targets and avoidance-related attributes shared the same response key); Overall difference score, Incompatible block—compatible block, i.e., a positive difference score indicates faster approach-related associations toward smoking-related pictures. Stroop: Score 1, RTs smoke card; Score 2, RTs neutral card; Overall difference score, Score 1—Score 2, i.e., a positive difference score indicates a greater interference for smoking-related stimuli.

### Stimulus response compatibility (SRC) task

As a first step, we examined the groups' error scores by means of a Univariate ANOVA. Results showed that the groups did not differ here: *F*_(3, 216)_ = 0.41, *p* = 0.75 (smokers: *M* = 0.06, *SD* = 0.04; non-smokers: *M* = 0.06, *SD* = 0.04; cravers: *M* = 0.06, *SD* = 0.04; ex-smokers: *M* = 0.07, *SD* = 0.05). Based on this error check, we excluded two participants from the analysis because their error percentage was greater than 20% (smokers: *n* = 1, ex-smokers: *n* = 1). To analyze the RT data, we subtracted RTs of the compatible block from RTs of the incompatible block. As such, a positive difference score indicates faster approach of smoking-related pictures. Next, we conducted a univariate ANOVA with Group (smokers, non-smokers, cravers, ex-smokers), Order SRC (compatible-incompatible, incompatible-compatible) and Order Tasks (one, two) as between-subjects factor, and the difference score as dependent variable. Of most interest here was the main effect of Group. Results showed that this effect was marginally significant, *F*_(3, 202)_ = 2.24, *p* = 0.085, eta^2^ = 0.03. However, *post-hoc* Bonferroni tests revealed that none of the between-group comparisons were significant (*p's* > 0.05). As such, the groups did not differ in their approach-avoidance responses toward smoking-related and control pictures (for an overview of means, standard deviations and *n*'s per group, see Table 2).

### Single target implicit association test (STIAT)

As a first step, we examined the groups' error score by means of a Univariate ANOVA. Results showed that the groups did not differ here: *F*_(3, 216)_ = 1.31, *p* = 0.27 (smokers: *M* = 0.04, *SD* = 0.03; non-smokers: *M* = 0.05, *SD* = 0.04; cravers: *M* = 0.04, *SD* = 0.04; ex-smokers: *M* = 0.04, *SD* = 0.04). Based on this error check, we excluded two participants from the analysis because their error percentage was greater than 20% (non-smokers: *n* = 1, ex-smokers: *n* = 1). To analyze the RT data, we subtracted RTs of the compatible block from RTs of the incompatible block. As such, a positive difference score indicates faster approach-related associations toward smoking-related pictures. Next, we conducted a univariate ANOVA with Group (smokers, non-smokers, cravers, ex-smokers), Order STIAT (compatible-incompatible, incompatible-compatible) and Order Tasks (one, two) as between-subjects factor, and the difference score as dependent variable. Of most interest here was the main effect of Group. Results showed that this effect was significant, *F*_(3, 202)_ = 5.74, *p* < 0.01, eta^2^ = 0.08. *Post-hoc* Bonferroni tests revealed the following: cravers vs. non-smokers: *p* < 0.001, cravers vs. smokers: *p* = 0.051, cravers vs. ex-smokers: *p* = 0.094. None of the other comparison reached significance (*p's* > 0.05). Following this, cravers had a stronger approach-bias toward smoking-related cues than smokers, non-smokers and ex-smokers (for an overview of means, standard deviations and *n*'s per group, see Table 2).

### Attentional bias

#### Emotional stroop

Prior to the analysis, a difference score was calculated per participant. Here, we only used RTs of the neutral card and the smoke card, not the practice card including the meaningless colored “XXX” strings. More precisely, RTs of the neutral card were subtracted from RTs of the smoke card. As such, a positive difference score indicates greater interference for smoking-related stimuli. We excluded 6 participants because their difference score deviated more than 3 SD from their group's mean difference score (non-smokers *n* = 2, cravers *n* = 4). We conducted a univariate ANOVA including the between-subjects factor Group (smokers, non-smokers, cravers, ex-smokers), Order Stroop Card (smoke-neutral, neutral-smoke) and Order Tasks (one, two), and the difference score as dependent variable. Of main interest here was the main effect of Group. However, results showed that this effect was not significant, *F*_(3, 192)_ = 0.4 *p* = 0.75. As such, there were no group differences concerning the interference of smoking-related vs. neutral stimuli (for an overview of means, standard deviations and *n*'s per group, see Table 2).

#### Ratings explicit attitudes toward smoking

Prior to analysis, scores on the eight adjective pairs were collapsed into one overall score. Here, higher sum scores signal a more negative attitude toward smoking. To investigate whether the groups differed concerning their explicit attitude toward smoking, a univariate ANOVA was conducted with Group (smokers, non-smokers, cravers, ex-smokers) as between-subjects factor and the collapsed attitude sum score as dependent variable. Results showed a significant main effect of Group, *F*_(3, 217)_ = 52.32, *p* < 0.001, eta^2^ = 0.42. *Post-hoc* Bonferroni tests revealed significant results for all group comparisons (*p's* < 0.01), except for the smoker vs. craver comparison, *p* = 0.1 (smokers: *M* = 33.66, *SD* = 4.47; non-smokers: *M* = 45.7, *SD* = 6.65; cravers: *M* = 33.82, *SD* = 5.68; ex-smokers: *M* = 38.11, *SD* = 6.41). Moreover, one sample *t*-tests showed that all four group means deviated significant from zero, *p's* < 0.001. This result pattern generally shows that non-smokers have the most negative attitude toward smoking, followed by ex-smokers, smokers, and cravers.

#### Correlations

Across the group of smokers, cravers, and ex-smokers, correlations were calculated for the following measures: AAT, SRC, STIAT, Stroop, explicit attitudes toward smoking, daily smoking, FTND scores, urge pre study, urge post-study. Table 3 gives an overview of these findings. We particularly expected to find positive correlations between tobacco-related approach and attentional biases. However, there were only two marginally significant correlations, i.e., between the AAT and the STIAT (*r* = 0.15), showing that the stronger the approach bias toward smoking-related pictures on the AAT, the stronger the approach-associations toward smoking-related words on the STIAT; and between the SRC and the Stroop (*r* = 0.15), showing that the stronger the approach bias toward smoking-related pictures on the SRC, the greater the smoking-related attentional bias on the Stroop. When looking at the correlations including explicit attitudes toward smoking, daily smoking, FTND scores, urge pre study, urge post-study, we found the following: Both the AAT and the SRC correlated significantly with explicit smoking attitudes (*r* = −0.15, marginally significant, and *r* = −0.18), showing that the stronger the approach bias toward smoking-related pictures, the less negative participants' attitude toward smoking was. Moreover, there was a marginally significant correlation between the Stroop and FTND scores (*r* = 0.16), indicating that the greater the smoking-related attentional bias on the Stroop, the higher participants' levels of nicotine dependence. Urge assesed before and after the computer tasks correlated with the STIAT (urge pre: *r* = 0.27, urge post: *r* = 0.25), and the Stroop (urge post: *r* = 0.17), showing that the higher levels of urge, the stronger the approach-associations toward smoking-related words on the STIAT, and the stronger the smoking-related attentional bias on the Stroop. Please note, however, that most of the correlations would not remain significant after controlling for multiple comparisons.

**Table 3:** **Correlations between AAT, SRC, STIAT, Stroop, explicit attitudes, daily smoking, FTND, urge pre-study, and urge post-study in smokers, cravers, and ex-smokers (*N* = 226)**.

	**1**	**2**	**3**	**4**	**5**	**6**	**7**	**8**
1. AAT	−							
2. SRC	−0.05	−						
3. STIAT	0.15^#^	0.07	−					
4. Stroop	−0.04	0.15^#^	−0.02	−				
5. Explicit attitudes	−0.15^#^	−0.18^*^	−0.13	−0.07	−			
6. Daily smoking	0.06	−0.02	0.05	−0.05	0.14^#^	−		
7. FTND	−0.1	0.00	0.02	0.16^#^	−0.00	0.02	−	
8. Urge pre-study	0.07	0.09	0.27^***^	0.13	−0.19^*^	0.14^#^	0.24^**^	−
9. Urge post-study	0.12	0.1	0.25^**^	0.17^*^	−0.29^***^	0.07	0.28^**^	0.82^**^

AAT, Approach-Avoidance Task (difference score smoke-related pictures—difference score control pictures); SRC, Stimulus Response Compatibility (incompatible—compatible); STIAT, Single Target Implicit Association Test (incompatible—compatible); Daily smoking, cigarettes per day; FTND, Fagerström Test for Nicotine Dependence.

# p < 0.100,

* p < 0.05,

** p < 0.01,

*** p < 0.001.

## Discussion

The present study examined the role of approach and attentional biases in tobacco dependence. We tested four groups, namely smokers, cravers, ex-smokers, and non-smokers. The following tasks were employed: To assess approach-related biases, we used the Approach Avoidance Task (AAT), the Stimulus Response Compatibility Task (SRC), and a Single Target Implicit Association Test (STIAT). A modified Stroop including smoking-related words was administered to assess attentional biases. Moreover, we assessed explicit attitudes toward smoking and levels of craving over the course of the study. We expected to find strong tobacco-related approach and attentional biases in smokers and cravers compared to ex-smokers and non-smokers. However, the general pattern of results did not confirm these expectations. Approach responses assessed during the AAT and SRC did not differ between groups. Moreover, the Stroop did not show the expected interference effect. Regarding the data of the STIAT, results were partly in line with our expectations: Cravers showed stronger approach associations toward smoking-related cues, whereas non-smokers showed stronger avoidance associations. However, no such differences in approach-avoidance associations were found in smokers and ex-smokers. Generally, correlational analyses did not reveal the expected positive correlations between tobacco-related approach and attentional biases among smokers, cravers, and ex-smokers. However, we did find some patterns that are in line with the theory, e.g., the stronger the approach bias toward smoking-related pictures, the stronger the smoking-related approach associations. Regarding the assessment of participants' explicit smoking-related attitudes, results were generally indicative of a negative attitude toward smoking, with non-smokers and ex-smokers having the most negative attitudes toward smoking. Finally, results showed that smokers' and cravers' level of craving significantly increased over the course of the study. In the group of ex-smokers, this increase was marginally significant.

To summarize, our data do not provide strong evidence for the role of approach and attentional biases in tobacco dependency, except for findings on the STIAT. Given the large sample size of each group, a lack of statistical power does not seem to be a likely explanation. Hence, a closer inspection of the tested groups, the tasks and their stimuli could help to understand these null-findings. Regarding the groups, their average smoking behavior is the first index to check and compare. Across our groups, smokers, cravers, and ex-smokers smoked 12–13 cigarettes a day. These scores are rather low when comparing them, for example, with the samples tested by Wiers C. E. et al. (2013): In that study, smokers and ex-smokers reported an average between 22 and 24 cigarettes a day. Thus, one could argue that our groups were not “smoking enough” in order to show tobacco-related approach and attentional biases. However, other studies found such biases in samples that exhibited a similar smoking behavior than ours (e.g., Munafò et al., 2003; Bradley et al., 2004; Mogg et al., 2005). Moreover, in our study, smokers and cravers were supposed to be active smokers for at least two years. Another index, i.e., the groups' score on the Fargerström Test of Nicotine Dependence (FTND), is also rather inconclusive. Our groups scored around five on the FTND, which does not deviate much from the values in other studies (e.g., Munafò et al., 2003; Bradley et al., 2008; Wiers C. E. et al., 2013). To conclude, the sample's general smoking-related characteristics match with other studies, and thus do not provide a sufficient explanation for the null-findings. Regarding the tasks we employed, we used well-established tasks in the context of tobacco-related approach and attentional biases (i.e., AAT, SRC; STIAT, and emotional Stroop). The AAT is a rather novel task for this specific type of addictive behavior. However, it has been proven successful in the assessment of alcohol-related approach biases (e.g., Palfai and Ostafin, 2003; Wiers et al., 2010, 2011; Eberl et al., 2013; Kersbergen et al., 2015). Therefore, given the fact we tapped into similar processes (i.e., approach biases), in combination with the successful results reported by the three previous studies (Wiers C. E. et al., 2013; Larsen et al., 2014; Machulska et al., 2015), the AAT seemed a promising instrument. Only the STIAT provided results that partly supported our predictions. That is, we found stronger approach associations toward smoking-related cues in cravers, whereas stronger avoidance associations were found in non-smokers. From a theoretical perspective, this is in line with assumptions put forward by dual process models of addiction (e.g., Deutsch and Strack, 2006; Wiers et al., 2007) and the incentive-sensitization model (Robinson and Berridge, 1993, 2003, 2008): For cravers who were deprived of smoking, smoking-related cues had a high incentive salience, which in turn automatically elicited an approach association. For non-smokers, in contrast, for whom smoking-related cues did not have incentive salience and were rather associated with negativity and unpleasantness, smoking-related cues automatically elicited an avoidance association. Interestingly, however, our STIAT version slightly deviated from that of other STIATs as it included a high number of trials. To summarize, the details of the specific tasks used cannot explain the present null-results. Finally, the choice of stimulus material needs to be analyzed. The AAT and SRC included pictures that depicted clear smoking-related scenes or attributes, and the corresponding matched control picture. A problem with such matched control pictures could be that they were in fact “too good.” That is, given their high similarity with the smoking-related pictures, they were possibly not distinctive enough. This, in combination with participants' instruction to react to the pictures as quickly as possible, could be partly responsible for not finding any differences in approach-related response within the tested groups. Moreover, some of the pictures contained food-related objects, so the control pictures could have elicited approach tendencies too. In this context, Machulska et al. (2015) suggest that it might be beneficial to use pictures that depict the commencement of smoking behavior (following findings by Stippekohl et al., 2012, and for similar reason when using pleasant vs. unpleasant smoking-related pictures, see Bradley et al., 2008). Our picture set included only three of such pictures, which could partly explain the null-findings of the AAT and SRC. Finally, there are three additional limitations that could partly explain the present findings. A first limitation is the low reliability of some of the tasks we applied. Second, we did not use baseline CO levels as an inclusion criterion. Third, we cannot rule out that the smokers who were asked to smoke a cigarette prior to testing experienced a smoking-related priming effect during testing. Especially the latter two issues could have affected the results in an unfortunate manner.

To conclude, although the study has some limitations as highlighted above, the present findings remain rather puzzling. Our results neither replicate earlier findings, nor support predictions of dual process models of addiction (e.g., Deutsch and Strack, 2006; Wiers et al., 2007) or the incentive-sensation model (Robinson and Berridge, 1993, 2003, 2008). Following this, our findings do not provide support for studies aiming to re-train approach and attentional biases, a development which has revealed promising findings in the area of alcohol addiction. Here, results showed that computerized trainings, i.e., procedures derived from “Cognitive Bias Modification” techniques (cf. Koster et al., 2009; Woud and Becker, 2014), are able to reduce alcohol-related approach biases (e.g., via Alcohol-AAT-Training, AAATT). Most important, however, results showed that such trainings improve treatment outcomes even at one-year follow- up (Wiers et al., 2010, 2011; Eberl et al., 2013; Gladwin et al., 2014; and for an overview of CBM-related results in addiction, see Wiers R. W. et al., 2013). In fact, CBM training could be also quite useful in the context of tobacco-related biases, as they operate comparably to those reported in the alcohol literature. Indeed, one published study applied a computerized re-training in the context of tobacco dependence (Wittekind et al., 2014). This study found that tobacco avoidance training reduced levels of cigarette consumption and dependence. However, the study is only a pilot study without a control group. Hence, these data should be interpreted with caution.

Following our null-findings, we suggest that future research should address a number of issues. To start with, studies should further examine the exact conditions of tobacco-related approach and avoidance biases. Moreover, this has to be examined among various relevant groups, e.g., smokers, cravers, ex-smokers, and non-smokers, while taking craving into account (Watson et al., 2013). Although the empirical evidence is rather supportive of the existence of tobacco-related biases, a few studies also found results that do not support a strong role of such biases in smoking. To illustrate, Munafò et al. (2003) and Mogg and Bradley (2002) did not find differences in information processing biases between abstinent smokers and non-abstinent smokers, whereas Larsen et al. (2014) did not find differences in biases between smokers and non-smokers. Hence, it might be possible that there are a number of subtle, boundary conditions, which are not yet fully understood.

Taken together, we did not find the expected tobacco-related approach and attentional biases, and therefore encourage future research to advance our understanding of the nature of these phenomena.

### Conflict of interest statement

The authors declare that the research was conducted in the absence of any commercial or financial relationships that could be construed as a potential conflict of interest.

## Footnotes

^1^Please note that there are additional missing data for the self-report measures and reaction time data due to technical problems during testing.

^2^For the sake of clarity, we only report the outcome of main interest in the text. Hence, please find the additional outcomes here (i.e., main effects and interactions) of the analyses of the AAT, SRC, STIAT and Stroop data: AAT: Order Tasks, *F*_(1, 206)_ = 0.25, *p* = 0.62, Group × Order Tasks, *F*_(3, 206)_ = 0.89, *p* = 0.45. SRC: Order Tasks, *F*_(1, 202)_ = 0.49, *p* = 0.49, Order SRC, *F*_(1, 202)_ = 0.41, *p* = 0.52, Group × Order Tasks, *F*_(3, 202)_ = 1.59, *p* = 0.19, Group × SRC order, *F*_(3, 202)_ = 2.88, *p* = 0.04, eta^2^ = 0.04, Order Tasks × SRC Order, *F*_(1, 202)_ = 0.02, *p* = 0.9, Group × Order Tasks × SRC Order, *F*_(3, 202)_ = 0.59, *p* = 0.63. STIAT: Order Tasks, *F*_(1, 202)_ = 4.1, *p* = 0.04, eta^2^ = 0.02, STIAT Order, *F*_(1, 202)_ = 1.1, *p* = 0.3, Group × Order Tasks, *F*_(3, 202)_ = 0.64, *p* = 0.59, Group × STIAT Order, *F*_(3, 202)_ = 4.43, *p* < 0.01, eta^2^ = 0.06, Order Tasks × STIAT Order, *F*_(1, 202)_ = 18.62, *p* < 0.001, eta^2^ = 0.08, Group × Order Tasks × STIAT Order, *F*_(3, 202)_ = 4.13, *p* < 0.01, eta^2^ = 0.06. Stroop: Order Tasks, *F*_(1, 192)_ = 0.56, *p* = 0.45, Order Stroop, *F*_(1, 192)_ = 14.32, *p* < 0.001, eta^2^ = 0.07, Group × Order Tasks, *F*_(3, 192)_ = 0.53, *p* = 0.67, Group × Order Stroop, *F*_(3, 192)_ = 3.87, *p* = 0.01, eta^2^ = 0.06, Order Tasks × Order Stroop, *F*_(1, 192)_ = 0.48, *p* = 0.49, Group × Order Tasks × Order Stroop, *F*_(3, 192)_ = 2.51, *p* = 0.6, eta^2^ = 0.04.
