# Supplementary figures and images for: Assessment of Tobacco-Related Approach and Attentional Biases in Smokers, Cravers, Ex-Smokers, and Non-Smokers
Source: Front Psychol. 2016 Feb 26;7:172. doi: 10.3389/fpsyg.2016.00172 (PMC4767899; doi:10.3389/fpsyg.2016.00172)

Appendix

Example pictorial stimuli for AAT and SRC

| Smoke pictures | Matched control pictures |
| --- | --- |
| 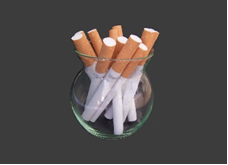 | 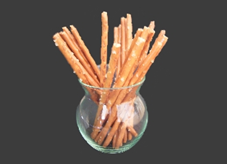 |
| 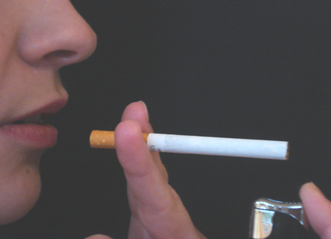 | 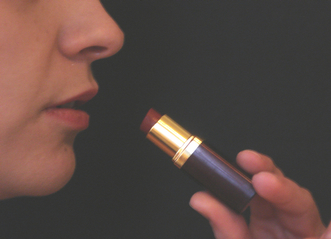 |
| 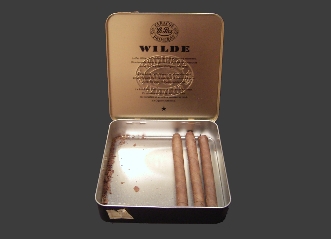 | 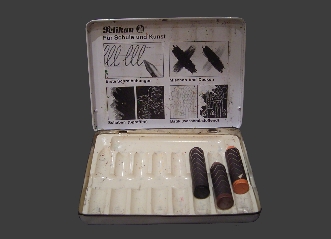 |
| 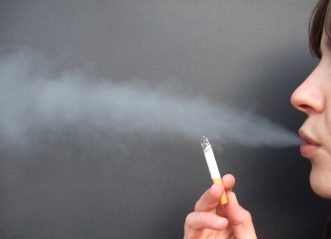 | 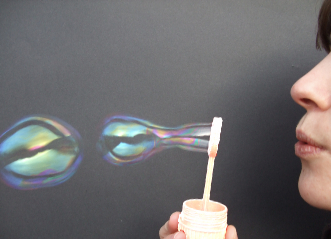 |

Supplement: Supplementary file 1 [file DataSheet1.docx]
